# Can Sensory- and Attachment-Informed Approaches Modify the Perception of Pain? An Experimental Study

**DOI:** 10.1155/2021/5527261

**Published:** 2021-11-11

**Authors:** Pamela Joy Meredith, Nicole Emma Andrews, Jessica Thackeray, Sophie Bowen, Cory Poll, Jenny Strong

**Affiliations:** ^1^School of Health and Behavioural Sciences, University of the Sunshine Coast (USC), Locked Bag 4, Maroochydore BC 4558, Queensland, Australia; ^2^Occupational Therapy, School of Health & Rehabilitation Sciences, The University of Queensland (UQ), Saint Lucia 4072, Queensland, Australia; ^3^Department of Occupational Therapy, Royal Brisbane and Women's Hospital, Metro North Hospital and Health Service, Herston 4006, Queensland, Australia; ^4^RECOVER Injury Research Centre, The University of Queensland (UQ), Herston 4006, Queensland, Australia; ^5^Tess Cramond Pain and Research Centre, Royal Brisbane and Women's Hospital, Metro North Hospital and Health Service, Herston 4006, Queensland, Australia

## Abstract

Accumulating evidence linking pain with both attachment and sensory processing variables introduces the possibility that attachment- and sensory-informed strategies may modify pain experiences. The aim of this study was to investigate this proposition using an experimentally induced pain procedure. Pain perceptions of individuals using either a sensory-informed (weighted modality) or an attachment-informed (secure base priming) coping strategy were compared with those of individuals using no designated coping strategy. An independent measures experimental study design was used with a convenience sample of 272 pain-free adults. Experimental participants (*n* = 156) were randomly allocated to either an attachment (*n* = 75) or a sensory (*n* = 81) intervention group. Data from these participants were compared to those of 116 participants involved in an earlier cold pressor study in which no coping strategy was used. All participants completed the same sensory, attachment, and distress questionnaires and participated in the same cold pressor pain test. ANCOVAs revealed that participants in the sensory- and attachment-informed intervention groups reported significantly higher pain thresholds than the control group. Participants allocated to the sensory group also reported higher pain intensity scores than the control group. There were no significant differences in pain tolerance between the three groups after controlling for covariates. While further research is required, findings encourage further consideration of sensory- and attachment-informed strategies for people anticipating a painful experience.

## 1. Introduction

Studies have linked adult *attachment* patterns with both experimental [1–3] and chronic [4–11] pain variables. With mounting evidence that attachment and sensory variables are meaningfully related [12], *sensory* patterns have also been linked with pain variables [13, 14] and studies have considered *both* sensory and attachment variables in relation to pain [12, 15]. As a first step in investigating potential clinical applications of attachment and sensory theories in the field of chronic pain [12, 15], the present study investigated perceptions of cold pressor-induced pain held by people using attachment- or sensory-informed coping strategies to those of people using no designated coping strategy.

### 1.1. Theoretical Background

This study draws on both attachment theory (see [16]) and the theory of sensory processing [17, 18] to develop and test two brief interventions in the context of acute pain. In the attachment literature, attachment insecurity has been associated with emotions and behaviours that have negative health impacts [19] including development of, and poorer recovery from, chronic pain [5, 6]. In contrast, securely attached people are more able to tolerate and manage stressful situations [1, 20], including pain [5, 6]. Based on attachment theory, individuals are most likely to perform well when they feel *safe* [16, 21]. A sense of safety can be elicited by a process called secure base priming whereby one imagines a loving and comforting experience. Kobak and Bosmans [22] noted that priming interventions involve preconscious or automatic levels of processing that do not require a change of internal working model to effect change in overall feelings of security. While suggested as a strategy to support individuals in pain [21, 23], only one study has investigated links between secure base priming and perceptions of pain. Rowe et al. [24] found that both secure and anxious primes, delivered during a cold pressor task, resulted in higher pain tolerance and threshold compared to neutral primes, but did not affect pain intensity or catastrophizing.

The theory of sensory processing recognizes that sensory stimuli affect our emotional states [17]. Approaches using sensory stimuli to improve self-regulation, known as sensory modulation approaches [18], have received growing attention in mental health and trauma fields [25]; however, there is little research specific to pain. A recent cold pressor study revealed that people who were more sensitive to stimuli (i.e., lower thresholds) were more likely to use maladaptive coping strategies, while those who were less sensitive (i.e., higher thresholds) reported more adaptive coping strategies [14]. Other studies have found that weighted sensory items providing deep pressure can produce a calming response [26, 27], including reducing pain and anxiety in adults during a dental procedure [28].

### 1.2. The Present Study

At present, evidence supports associations between sensory and attachment variables [14], and between these variables and perceptions of pain [2, 5, 6, 12–14, 20]. While a small amount of evidence suggests that coping strategies informed by these theories may diminish the impact of a painful experience, this research is in its infancy. In this study, the potential clinical applications of attachment and sensory theories in the field of chronic pain is considered, specifically, the impact of sensory- and attachment-informed coping strategies in an experimental pain task. We addressed the research question: do participants using either a sensory (weighted modality) or attachment (secure base priming) coping strategy vary in terms of pain threshold, tolerance, or pain intensity, compared to controls, during an experimentally induced pain procedure? Improving our understanding of these factors may assist in developing effective strategies to support management of painful procedures and, potentially, chronic pain.

## 2. Methods

### 2.1. Research Methods

This study utilized an independent measures experimental design in which different participants were involved in three different experimental conditions. This study received ethical approval from the institutional review board of The University of Queensland (no. 2018000282).

### 2.2. Subjects and Procedure

This study utilized a convenience sample of 272 pain-free participants. The intervention group (*n* = 156) were 47 men and 109 women, aged between 18 and 86 years (*M* = 33.7). These participants were randomly allocated to either an attachment (*n* = 75) or sensory (*n* = 81) intervention group. Comparison data was obtained from 116 healthy participants who participated in a previous study [6, 14], in which an identical experimental protocol was used without provision of a sensory or attachment coping strategy.

Participants met the following inclusion criteria: (1) English speaking; (2) 18 years old or above; (3) not currently experiencing pain; (4) no history of cardiovascular disease, diabetes, or Raynaud's disease; and (5) no cognitive or developmental disabilities. Participants were volunteers who received no incentives for participation. As seen in Table 1, most participants were Caucasian, married, had completed a bachelor's degree, and were employed full-time.

Data for the two experimental groups were collected in 2018 by three of the authors (S.B., C.P., and J.T.) in two Australian states. Invitations to participate in the study were extended by word-of-mouth and private messaging on social media. People who expressed interest in participating in the study were provided with an information sheet and consent form and screened to ensure they met inclusion criteria. Comparison group data were collected earlier in 2013-2014, with ethical approval by The University of Queensland's Behavioural and Social Science Ethical Review Committee (no. 2012000013) [6, 14].

After providing written consent, participants were asked to complete the questionnaire and were allocated to either attachment or sensory experimental conditions. Simple randomisation was used whereby participants chose a number (1 or 2) from a hat prior to the cold pressor task. Participants allocated to the sensory-informed coping strategy were provided with a weighted modality (2.5 kg weighted blanket), which was placed over their shoulders for the duration of the cold pressor task. Participants allocated to the attachment-informed coping strategy were trained in a secure base priming strategy, in which they were instructed to imagine a loving and comforting experience with a significant other. The cold pressor task was then conducted in a mutually convenient space following standard instructions [7]. Although specific testing locations varied across researchers and participants, all were private and quiet, and the same cold pressor apparatus and procedures were used with all participants. The comparison group also utilized the same written questionnaires and identical cold pressor protocol.

### 2.3. Measures

#### 2.3.1. Demographic Variables

Data was collected on participant age, gender, ethnicity, marital status, education level, and employment status.

#### 2.3.2. Adolescent/Adult Sensory Profile (AASP)

The AASP [29] is a 60-item self-report tool based on Dunn's [17] Model of Sensory Processing. The items are divided equally among four sensory processing patterns: low registration, sensation seeking, sensory sensitivity, and sensation avoiding. Questions are scored on a 5-point Likert scale (1, *almost never,* to 5, *almost always*). Internal consistency for each of the subscales has been shown to be satisfactory, with coefficient alpha (*α*) values of 0.82 for low registration, 0.79 for sensation seeking, 0.81 for sensory sensitivity, and 0.66 for sensation avoiding [30]. In the present study, the internal consistency based on the whole sample was good for low registration, sensory sensitivity, and sensory avoiding (*α* = 0.74, 0.73, and 0.73, respectively), and adequate for sensory seeking (*α* = 0.68).

#### 2.3.3. Experiences in Close Relationships-Revised Questionnaire Short Form (ECR-SF)

The ECR-SF [31] is designed to assess one's general pattern of adult attachment through self-report responses to questions about attachment anxiety and avoidance. The 12 questions are scored on a Likert scale (1, *not at all true*, to 7, and *very true*). Previous analyses of internal consistency reported *α* scores from 0.77 to 0.86 for the anxiety subscale, and 0.78 to 0.88 for the avoidance subscale [31]. In the present study, internal consistency was good for the anxiety (*α* = 0.73) and avoidance (*α* = 0.76) subscales.

#### 2.3.4. Depression Anxiety Stress Scale (DASS-21)

The DASS-21 [32] is a 21-item self-report measure of a person's stress, anxiety, and depression. Each item is scored on a four-point Likert scale (0, *did not apply to me at all*, to 3, *applied to me very much*, *or most of the time*) in the past seven days. Internal consistency of experimental groups for each of the subscales is reported to be good, with *α* scores of 0.88 for depression, 0.82 for anxiety, and 0.90 for stress [33]. In the present study, internal consistency based on the whole sample for each of the subscales was 0.81 (depression), 0.66 (anxiety), and 0.82 (stress).

#### 2.3.5. Cold Pressor Apparatus

The cold pressor apparatus has been widely used in pain-research to induce temporary hand and forearm pain [34]. The apparatus consists of an insulated container filled with water that is maintained at a temperature between 0° and 2°C. Participants are instructed to submerge their nondominant hand and forearm into the water for as long as possible or until the pain is unbearable [35]. Three pain variables, threshold, tolerance, and intensity, are recorded throughout the task. To measure *threshold,* participants are asked to report when they first feel what they would call pain. *Tolerance* is the length of time, in seconds, participants hold their hand in the iced water. To measure the individual's *pain intensity*, participants are asked to rate their level of discomfort on a 100-point scale in 20-second intervals. From this, mean pain intensity scores are calculated for each participant. A four-minute time limit is imposed on the cold pressor task (of which participants are not informed) to minimise risk [34]. The procedure, in its entirety, takes approximately 30 minutes.

### 2.4. Statistical Analysis

Statistical analyses were performed using IBM SPSS statistics for Windows (Version 25; IBM Corporation, Armonk, NY). Data screening was conducted to check for normality, identify outliers, and assess for missing data. Although outliers were identified, none were beyond parameters of the measures of the study; hence, they were retained. Missing data resulted in slightly decreased participant numbers for some analyses.

To identify potential confounding variables, all three groups (two experimental and one comparison) were compared on demographic and study variables using chi-square analyses or ANOVA, depending on the nature of the variables. In addition, all possible associations between continuous and categorical variables were examined using Pearson's correlation coefficients or ANOVA.

To test research questions, three ANOVAs were conducted with group membership (sensory-informed, attachment-informed, and comparison groups) as the independent variable and one of three pain variables (mean intensity ratings, threshold, or tolerance) entered into each model as dependent variables. Welch's ANOVA statistics were used for pain threshold as the data was found to violate the assumption of homogeneity. Attachment anxiety and attachment avoidance were added to each model and analysed using a series of ANCOVAs. In addition, variables that differed between groups, or were significantly associated with a pain variable, were also entered and analysed to control for potential cofounding variables in these ANCOVA models. Variance inflation factor (VIF) values were produced, and residuals were checked for linearity and normality for the final models [5].

## 3. Results

### 3.1. Preliminary Analyses

Participant demographics according to experimental group allocation are displayed in Table 1. Significant differences among the two experimental and comparison groups were identified for relationship status (*χ*^2^ (4, *N* = 269) = 17.50, *p*=0.002), education level (*χ*^2^ (8, *N* = 270) = 22.92, *p*=0.003), and employment status (*χ*^2^ (6, *N* = 270) = 29.72, *p* < 0.001). In addition, significant differences among the experimental and comparison groups were identified for the variables of age (*F* (2, 268) = 9.78, *p* < 0.001), stress (*F* (2, 267) = 11.97, *p* < 0.001), depression (*F* (2, 267) = 4.34, *p*=0.01), and attachment anxiety (*F* (2, 266) = 22.11, *p* ≤ 0.001) (see Table 2). These covariates were retained in each ANCOVA model.

Several experimental variables were significantly associated with pain outcomes. Sensory avoidance was significantly correlated with pain threshold (*r* (263) = .15, *p*=0.02). Gender was significantly associated with pain tolerance (*F* (1, 270) = 28.00, *p* < 0.001) and mean pain intensity scores (*F*(1,269) = .29, *p*=0.01). Sensory avoidance and gender were, therefore, included as covariates in ANCOVA models. Results of ANCOVA models, displayed in Table 3, are summarised below. Residual and scatter plots indicated the assumptions of normality and linearity were satisfied for all ANCOVA models. All VIF values were <2, indicating that multicollinearity was not present in the final models.

### 3.2. Pain Threshold

A significant difference was found between the three groups for the pain threshold variable using ANOVA (Welch's *F* (2, 110) = 15.3, *p* < 0.001). This significance was maintained while controlling for age, stress, depression, attachment anxiety, attachment avoidance, sensory avoiding, gender, relationship status, education level, and employment status, using ANCOVA (*F* (2, 239) = 8.67, *p* < 0.001). Participants allocated to the sensory-informed coping strategy (*M* = 48.55) and the attachment-informed coping strategy (*M* = 64.84) had pain thresholds significantly higher (*p*=0.005 and *p* < 0.001, respectively) than participants in the comparison group (*M* = 18.13). None of the covariates were significant in this model.

### 3.3. Pain Intensity

A significant difference was found between groups for the pain intensity variable using ANOVA (*F* (2, 270) = 3.69, *p*=0.03). This difference was maintained when controlling for age, stress, depression, attachment anxiety, attachment avoidance, gender, relationship status, education level, and employment status using ANCOVA (*F* (2, 241) = 3.89, *p*=0.02). Participants allocated to the sensory group reported significantly higher pain intensity scores than participants in the comparison group (*M* = 56.87 versus *M* = 47.05, *p*=0.01). None of the covariates were significant in this model.

### 3.4. Pain Tolerance

Comparison between groups for pain tolerance did not reach significance using ANOVA (*F* (2, 243) = 0.03, *p*=0.97). Gender was found to explain variance in participant scores for pain tolerance using ANCOVA (*F* (1, 242) = 15.65, *p* < 0.001). Male participants demonstrated significantly higher pain tolerance (*M* = 209.38) compared to female participants (*M* = 164.16, *p* < 0.001).

## 4. Discussion

The aim of the present study was to determine whether participants using either a sensory-informed (weighted modality) or attachment-informed (secure base priming) coping strategy would differ from those using no strategy in terms of their pain threshold, tolerance, and average pain intensity, during an experimentally induced pain procedure. Developing a better understanding of how these coping strategies impact the perception of pain may inform clinical approaches for people experiencing pain.

Consistent with theoretical expectations, participants allocated to both the sensory- and attachment-informed coping strategies reported a higher *pain threshold* than participants who used no coping strategy. This finding was robust, and not impacted by control variables. It is consistent with the small body of literature that has revealed that primed security [24, 36] and sensory-informed coping strategies, such as deep pressure [26, 28], can influence perceptions of, or responses to, painful experiences. Rowe et al. [24] (p. 500) suggested that attachment priming “…induced lower pain sensitivity due to the pain inoculating effect of having activated representations of positive relationship experiences regarding care in times of distress.” The shift of the autonomic nervous system from a sympathetic response to a parasympathetic response, resulting in improved arousal modulation and increased calm, has been proposed to explain such findings [28]. Based on findings of our study, individuals may benefit from utilising either a sensory- or attachment-informed coping strategy when anticipating a painful experience.

In contrast to results for pain threshold, participants allocated to the sensory group reported significantly higher *pain intensity* scores than participants in the control group, and this relationship was maintained when control variables were included. This study is not the first to find that a therapeutic intervention contributed to perceptions of increased pain intensity (e.g., see psychotherapy [37]). While explanations remain speculative, this finding may be linked to attentional factors. Arguably, application of a weighted modality could draw attention either towards the body or away from the painful experience. Distracting attention *away* from a painful experience has been shown to modulate acute pain [38], resulting in expectations that, together with the calming effect of the proprioceptive input, the weighted modality would decrease pain intensity. In contrast, experiencing sensory stimuli in two parts of the body (forearm/hand and shoulders) while engaged in a pain-inducing (alerting) activity may draw attention towards the body, resulting in increased perceptions of threat and pain intensity for some individuals. This complex association warrants further empirical attention.

Use of the attachment-informed coping strategy was not associated with perceptions of pain intensity in the present study. This is consistent with previous findings by Rowe et al. [24], although they provided no explanation for this lack of association. Theoretically, secure base priming should activate representations of secure, caring relationship experiences, lowering distress levels, and therefore decreasing sensitivity to pain [24]. It is possible that most participants in the present pain-free sample were not especially distressed by the cold pressor task, minimizing the relative impact of this intervention. This finding warrants further attention including replication of the present study and investigation of the strategy with people who have painful medical conditions.

There were no differences between the mean *pain tolerance* scores for the control, sensory-informed, or attachment-informed coping strategy groups. These findings are at variance with expectations derived from previous studies discussed earlier in the paper. For example, Rowe et al. [24] found that use of a security prime resulted in higher tolerance compared to the control group. Similarly, use of sensory-informed strategies, such as weighted blankets, has been effective in preventing and managing dysregulated states for children, adolescents, and the elderly [39]. Reasons for the lack of a significant result for either strategy in our study can only be speculated. In terms of measuring pain tolerance, the relatively high proportion of participants (58%) reaching the four-minute upper time ceiling imposed on the cold pressor task in this study may have impacted the significance of these results. While the four-minute time limit has conventionally been used as a safety measure in this experimental pain paradigm, a recent paper has suggested that a five-minute ceiling is within the safety parameters of a cold pressor task [34]. This may be considered in further research.

While it is not the focus of this study, gender was identified as the only variable associated with pain tolerance, with men reporting significantly higher pain tolerance compared to women. The association between pain tolerance and gender was not unexpected, with substantial literature revealing higher pain tolerance for men than women [40–42]. Various reviews seeking to explain these gender-related differences have identified numerous but inconsistent mechanisms, including perceptual ability and physiologic factors [42]: differences in physiology and hormones, temporal summation, allodynia, and secondary hyperalgesia, less efficient endogenous pain inhibitory systems, depression or anxiety, cognitive and social factors, and past individual history of pain [41]. There is still much to understand in relation to gender implications in pain.

### 4.1. Limitations and Future Research Directions

The results of this study should be interpreted cautiously. Although much published research has used convenience sampling [12], use of this method may introduce sampling bias. To minimise this bias, three researchers gathered data and were unaware of each participant's attachment and sensory processing classifications until after the cold pressor task had been completed. While efforts were made to randomly allocate participants, the groups were significantly different in key demographic and study variables, complicating comparisons. Importantly, this issue was addressed by controlling for these variables in analyses. The reliance on self-report measures is a further study limitation, with increased risk of social desirability bias and shared method variance. Consequently, future studies should consider including objective measures such as the Adult Attachment Interview [43] and measures of social desirability.

The pain threshold variable in this study violated the assumption of homogeneity of variance, likely due to the unequal sample sizes. As a result, the *F* statistic may have underestimated the significance of differences between participants in experimental and comparison groups [44]. To address this, future research may seek more equal group sizes, as well as within-subject repeated measures studies, to further investigate the impact of sensory- and attachment-informed approaches on pain intensity and pain tolerance.

The exploratory nature of this study means that the results require replication to improve confidence. Also, with evidence from a recent study that participants' pain sensitivity may be modulated differently in everyday life compared to laboratory environments [45], results may not be transferable to individuals experiencing pain outside an experimental paradigm. While studies using healthy volunteers and experimental pain paradigms can provide important preliminary data to inform research with clinical samples such as those who are experiencing pain [45], future research should engage participants with existing pain conditions.

### 4.2. Clinical Implications

Although further investigation of this topic is required, this study presents preliminary evidence that sensory- and attachment-informed coping strategies may modify the experience of pain. While more research is needed before clinical implications can be detailed with confidence, findings suggest that an individual experiencing acute pain, such as a dental procedure or immediately following an injury, may benefit from a sensory- or attachment-informed coping strategy.

## 5. Conclusion

The results of this study suggest that sensory- and attachment-informed coping strategies may contribute to a higher pain threshold, and that the use of a weighted modality may increase perceptions of pain intensity for people experiencing acute pain. These results contribute to a growing body of evidence that attachment and sensory theories may usefully inform clinical interventions for people experiencing pain. This knowledge may be utilized by clinicians working with people who are experiencing an acute pain episode.

## Figures and Tables

**Table 1:** Participant demographics according to experimental group allocation with chi-square analysis (*N* = 272).

Variable	Coping strategy groups				Comparison group *N* = 116		Chi-square value
	Sensory modality *n* = 81		Secure base priming *n* = 75				
	*n*	(%)	*N*	(%)	*n*	(%)	
Gender							3.83
Male	22	(27.2)	25	(33.3)	47	(40.5)	
Female	59	(72.8)	50	(66.7)	69	(59.5)	
Relationship status							17.50^*∗∗*^
Single/widower	37	(45.7)	21	(28.0)	64	(55.2)	
Married	31	(38.3)	32	(42.7)	26	(22.4)	
De facto	12	(14.8)	21	(28.0)	25	(21.6)	
Missing	1	(1.2)	1	(1.3)	1	(0.90)	
Educational level							22.92^*∗∗*^
Up to Grade 10	13	(16.0)	15	(20.0)	9	(7.8)	
Grade 12	19	(23.5)	15	(20.0)	48	(41.4)	
TAFE	10	(12.3)	14	(18.7)	22	(19.0)	
Undergraduate degree	24	(29.6)	19	(25.3)	31	(26.7)	
Postgraduate degree	14	(17.3)	11	(14.7)	6	(5.2)	
Missing	1	(1.2)	1	(1.3)	0	(0.0)	
Employment status							29.72^*∗∗*^
Full-time	35	(43.2)	40	(53.3)	54	(46.6)	
Part-time	22	(27.2)	14	(18.7)	20	(17.2)	
Retired/not employed	12	(14.8)	7	(9.3)	39	(33.6)	
Others	11	(13.6)	13	(17.3)	3	(2.6)	
Missing	1	(1.2)	1	(1.3)	0	(0.0)	

*Note.* TAFE = technical and further education.  ^*∗∗*^*p* < 0.01. Where subgroup numbers were <5, the variable was recoded to remove this category from analysis.

**Table 2:** Details of participant study variables presented according to coping strategy, compared using ANOVA, *N* = 272.

Variable	Sensory modality			Secure base priming			Comparison group			*F*
	*M*	SD	Range	*M*	SD	Range	*M*	SD	Range	
Age (years)	37.6	16.2	18–86	36.0	12.4	20–65	29.5	12.7	18–64	9.78^*∗∗*^
*DASS*										
Depression	3.6	5.7	0–24	3.0	4.0	0–20	5.3	6.3	0–38	4.34^*∗*^
Anxiety	4.6	6.6	0–32	4.2	4.3	0–18	5.1	5.1	0–26	0.74
Stress	7.2	7.1	0–30	6.2	5.0	0–22	11.1	8.6	0–40	11.97^*∗∗*^
*AASP*										
Low-reg	32.2	7.4	14–52	34.3	7.8	22–57	33.3	7.1	19–50	1.63
Seeking	46.6	8.7	2–66	47.5	6.7	28–61	48.8	7.8	24–66	2.00
Sensitivity	33.7	8.5	13–51	36.0	8.4	20–57	34.7	7.7	18–59	1.52
Avoiding	34.7	8.3	12–57	35.4	9.1	19–65	33.98	7.5	19–53	0.72
*ECR*										
Anxiety	19.2	5.8	6–34	20.7	6.22	6–34	14.9	6.6	6–33	2.11^*∗∗*^
Avoidance	15.9	7.2	6–35	15.3	5.97	6–33	17.1	6.5	6–33	1.91
Pain threshold (seconds)	55.1	72.3	1–240	63.8	81.65	2–240	23.6	19.5	0–110	15.26^*∗∗*^
Pain tolerance (seconds)	174.5	88.5	18–240	173.6	81.09	25–240	183.3	82.4	1–240	0.41
Average pain intensity (0–100)	55.5	24.3	7.1–100	47.3	22.0	4.7–95	47.4	21.8	0–90	3.69^*∗*^

*Note.* DASS = Depression Anxiety Stress Scale Questionnaire; AASP = adult/adolescent sensory profile; ECR = experiences in close relationship scale; *M* = mean; SD = standard deviation.

**Table 3:** *B* values and summary statistics for final three ANCOVA models for pain threshold, tolerance, and mean intensity, *N* = 272.

Variable	Dependent variables		
	Threshold	Tolerance	Mean intensity
*Coping strategy utilised*			
Sensory modulation	30.41^*∗∗*^	−1.81	9.83^*∗*^
Attachment	46.70^*∗∗*^	−4.64	1.70
Control	R	R	R
*Covariates*			
*Gender*			
Male	10.82	45.22^*∗∗∗*^	−5.51
Female	R	R	R
*Relationship status*			
Single/widower	17.12	1.89	0.13
Married	13.24	21.66	2.93
De facto	R	R	R
*Education level*			
Up to year 10	7.85	43.40^*∗*^	−8.33
Year 12	−4.10	9.63	0.72
TAFE	−8.70	22.79	−5.16
Undergraduate	16.04	29.86	−8.30
Postgraduate	R	R	R
*Employment*			
Full-time	8.98	−21.27	−5.15
Part-time	−2.50	−17.80	−5.71
Retired/not employed	−3.60	−15.09	1.16
Others	R	R	R
Age	0.54	−0.48	−0.14
Stress	0.64	−0.52	0.04
Depression	−0.79	−1.79	0.17
Attachment anxiety	−1.14	0.13	−0.11
Attachment avoidance	0.28	−0.37	−0.26
AASP-sensory avoiding	0.88	1.08	−0.13
*Summary statistics*			
*F*	8.67^*∗∗*^	0.05	3.89^*∗*^

*Note.* AASP = adult/adolescent sensory profile; R = reference category.  ^*∗*^*p* < 0.05,  ^*∗∗*^*p* < 0.01, and  ^*∗∗∗*^*p* < 0.001.

## Data Availability

The data are available upon request by contacting Pamela Meredith at pmeredith@usc.edu.au.
